# Protective Effect of Tetramethylpyrazine on Myocardial Ischemia-Reperfusion Injury

**DOI:** 10.1155/2014/107501

**Published:** 2014-07-24

**Authors:** Weidong Qian, Xingjiang Xiong, Zhuyuan Fang, Haiting Lu, Zhensheng Wang

**Affiliations:** ^1^Department of Cardiology, Traditional Chinese Medicine Hospital of Wujin District, Affiliated Hospital of Nanjing University of Chinese Medicine, Changzhou 213161, China; ^2^Department of Cardiology, Guang'anmen Hospital, China Academy of Chinese Medical Sciences, Xicheng District, Beijing 100053, China; ^3^Department of Cardiology, Jiangsu Province Hospital of Traditional Chinese Medicine, Jiangsu 210029, China

## Abstract

Myocardial ischemia-reperfusion injury (MIRI) is a common pathological and physiological phenomenon. Tetramethylpyrazine is the extract of the traditional Chinese medicine Chuanxiong, which can exert protective effects on MIRI in multiple ways. This paper reviewed the current research progress and evidence about the cardiovascular effects of tetramethylpyrazine, which included protecting mitochondria and improving energy metabolism, scavenging oxygen free radicals (OFRs) to inhibit lipid peroxidation, attenuating calcium (Ca^2+^) overload and maintaining Ca^2+^ homeostasis in cells, inhibiting apoptosis and protecting myocardial cells, interfering with the inflammatory reaction and mitigating cell injury, interfering with cell signaling pathways, and improving function of endothelial cells and protecting myocardial cells. However, further rigorously designed randomized controlled trials are warranted.

## 1. Introduction

**Figure 1:**
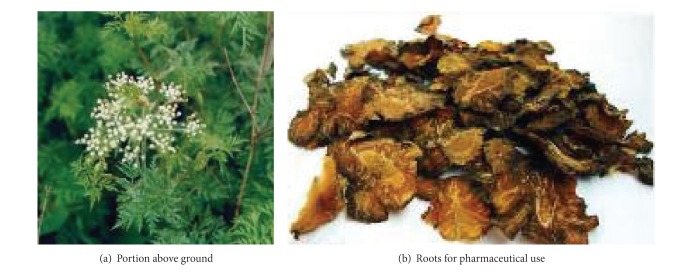
Morphology of tetramethylpyrazine.

**Figure 2:**
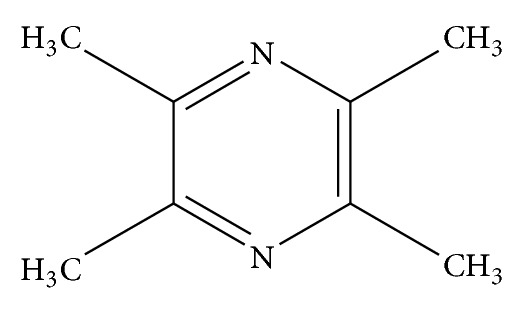
Molecular formula of tetramethylpyrazine.

Myocardial ischemia-reperfusion injury (MIRI) involves myocardial metabolic disorders and structural remodeling after reperfusion of the ischemic myocardium [1]. Previous studies have showed that the inflammatory response, platelet aggregation and microembolization, and cell death contributed significantly during the process of MIRI [2]. Conventional medicine therapy currently used in the treatment of MIRI includes nitrate, statins, Ca^2+^ antagonists, and angiotensin converting enzyme inhibitors (ACEI) [3]. However, undesirable effects of antianginal therapy do influence treatment adherence to a certain extent. A certain portion of patients with MIRI turned to traditional Chinese medicine therapy. Recent years have seen an increase in research relating to herbs for the treatment of MIRI, and tetramethylpyrazine (TMP) is among the most popular. TMP is an alkaloid found in the roots of* Ligusticum chuanxiong* Hort (LC; Umbelliferae) (as shown in Figures 1 and 2). TMP exerts a protective effect on MIRI in multiple ways with multiple targets, as described in this literature review.

## 2. Pharmacology

### 2.1. Protect Mitochondria and Improve Energy Metabolism

The heart requires a large amount of energy to maintain its normal physiological functions. Myocardial metabolic disorders have been reported to be involved in the pathogenesis of MIRI [4]. Myocardial ischemia reduces aerobic metabolism in the myocardium, and anaerobic metabolism becomes the main pathway. Anaerobic metabolism produces a large amount of acidic products, which in turn can induce intracellular acidic toxicity and thereby impair cell microstructure. Meanwhile, production of adenosine triphosphate (ATP) decreases rapidly, thereby reducing the mitochondrial activity of Ca^2+^-ATPase and Mg^2+^-ATPase. Therefore, mitochondrial Ca^2+^ levels are increased significantly. Disorders in energy metabolism can also induce mutations in myocardial genes and abnormal expression, thereby resulting in apoptosis [5].

Wang et al. reported that TMP can ameliorate MIRI by increasing energy production in myocardial cells [6]. A proposed mechanism is that TMP can reduce degradation of myocardial ATP and increase ATP generation. Through this pathway, energy storage in myocardial cells is increased, which could protect high-energy phosphate compounds in the myocardium.

Zhu et al. reported that Na^+^-K^+^-ATPase in myocardial tissues is not sensitive to ischemic injury but is sensitive to reperfusion injury [7]. TMP could protect the Na^+^-K^+^-ATPase activity of ischemic myocardial tissues after reperfusion. Shi et al., using molecular biological methods, observed that TMP could increase absorption of ^3^H-leucine and ^3^H-uridine under oxygen- and sugar-deficient conditions in myocardial cells [8]. TMP could also stimulate the synthesis of protein and RNA as well as increase expression of nitric oxide synthase in oxygen- and sugar-deficient myocardial cells to enhance their tolerance of these deficiencies.

Wang and colleagues found that TMP could significantly alleviate or prevent the swelling or degeneration of mitochondria, breakage and dissolution of myofilaments, and the swelling and damage of the sarcolemma during MIRI [9]. Their study revealed that TMP could protect the myocardium by maintaining the complete structure of biological membranes and myocardial fibers and reducing injury to mitochondria.

Based on a study of key respiratory enzymes of mitochondria* in vivo*, Wan and colleagues reported that TMP strongly antagonizes the reduction of activity of succinate dehydrogenase (SDH) and cytochrome oxidase (CCO) during MIRI [10]. Li et al. reported that the protective effect of TMP in a rat model of myocardial ischemic injury could be related to the increased activity of Ca^2+^-ATPase and Ca^2+^-Mg^2+^-ATPase and regulated expression of the Bcl-2 gene [11]. Wang et al. reported that TMP combined with L-arginine could improve mitochondrial function during MIRI by decreasing production of oxygen free radicals and reducing Ca^2+^ overload [12]. The effect of TMP on mitochondria protection and energy metabolism improvement during MIRI was shown in Table 1.

### 2.2. Scavenge Oxygen Free Radicals (OFRs) to Inhibit Lipid Peroxidation

Free radicals are generated under physiological conditions to maintain normal metabolism. High levels of free radicals are harmful to the body, and so they are scavenged to maintain a dynamic balance. Superoxide dismutase (SOD), glutathione peroxidase (GSH-Px), heat-shock protein (HSP)70, and H_2_O_2_ are essential protective substances in the myocardium. Galang et al. demonstrated that treatment using SOD and H_2_O_2_ can significantly reduce the apoptosis of myocardial cells in rat hearts during MIRI* in vitro* [13]. Free radicals are among the key components of MIRI [14]. OFRs can injure biological membranes, proteins, nucleic acids, chromosomes, extracellular-space components, and mitochondria during MIRI and induce myocardial injury [15].

Studies have shown that TMP can scavenge reactive oxygen species, regulate production of nitric oxide (NO), and prevent the formation of peroxynitrites [16]. TMP can strongly scavenge OFRs and has effects upon cell toxicity [17]. Liu and colleagues suggested that the potential cardioprotective mechanism of TMP should contribute (at least in part) to its prominent antilipid peroxidation and antifree radical-formation effects. Hence, it could protect the heart from lipid peroxidation-induced toxicity [18]. Wang et al. reported that TMP can protect the myocardium by activating SOD and GSH-Px and stimulating HSP70 mRNA and the corresponding protein expression [19]. Chen and colleagues reported that TMP could suppress ischemia-induced ventricular arrhythmias and reduce the infarct size resulting from ischemia-reperfusion injury* in vivo* [20]. This cardioprotective effect of TMP may be associated with its antioxidant activity* via* induction of the expression of heme oxygenase- (HO-) 1 and its capacity for neutrophil inhibition. Xu et al. reported that in a rabbit model of MIRI, serum levels of malonaldehyde (MDA) in the TMP group were reduced significantly, whereas levels of GSH-Px and GSH-Px lipid peroxide were increased considerably [21]. Therefore, TMP could inhibit OFR-induced damage to myocardial cells by protecting SOD activity, enhancing the scavenging of OFRs and reducing formation of lipid peroxides. Wan and colleagues reported that the activities of SOD, GSH-Px, Ca-ATPase, and Na-K-ATPase in myocardial cell membranes were higher in the TMP-protected group than in the control group, whereas levels of MDA and Ca^2+^ were much lower [22]. Also, mitochondrial activities of SOD and GSH-Px were increased appreciably, whereas that of MDA was reduced severely.

Zhang and colleagues reported that TMP could significantly protect myocardial function in a rat model of MIRI. The proposed mechanism was that it could inhibit free-radical generation and scavenge OFRs [23]. Gu et al. reported that TMP postconditioning could significantly protect against MIRI in rats [24]. They found that TMP postconditioning could reduce the prevalence of arrhythmias and infarct size. They proposed that the mechanism could be related to increases in the production of SOD, NO, and nitric oxide synthase (NOS) and reduction of MDA generation. Wan and colleagues found that TMP has strong effects against the reduction of activity of SDH and cytochrome oxidation in MIRI. They proposed a mechanism related to increased scavenging of OFRs and inhibition of lipid peroxidation [25].

Piao et al. observed ischemia-reperfusion in rabbit hearts* in vitro* with TMP in a cardioplegic solution. They found that in the TMP group, MDA level was reduced and SOD activity increased significantly [26]. The microstructure of myocardial tissues was injured mildly, and they concluded that TMP had potent protective and antioxidant activities on the ischemic myocardium. Based on a model of myocardial cells with hypoxia-reoxygenation injury, Zhang et al. studied the influence of TMP on lactate dehydrogenase (LDH) in myocardial nutrient solution and MDA in myocardial cells. They found that levels of LDH and TMP were decreased by TMP [27]. Zhou and Liu reported that TMP has a strong protective effect towards hypoxia-reoxygenation injury* in vitro* that could be related to inhibition and scavenging of free radicals by TMP [28].

Chen and colleagues reported that TMP preconditioning could significantly improve left-heart function and reduce infarct size and arrhythmia in a rat model of MIRI* in vitro*. It could also increase the myocardial activity of SOD and GSH-Px, reduce MDA levels, upregulate expression of HSP70, and induce potent delays in protective functions [29]. Qin and colleagues also demonstrated that TMP could significantly antagonize the reduction of activity of SOD and GSH-Px and the increase in MDA content in a rat model of MIRI [30]. Yue et al., using a hemorrhagic-shock model of reperfusion in rabbits, found that serum levels of MDA were lowered dramatically and that the whole-blood activity of SOD and GSH-Px was significantly increased after TMP reperfusion [31]. The effect of TMP on scavenge OFRs to inhibit lipid peroxidation was shown in Table 2.

### 2.3. Attenuate Calcium (Ca^2+^) Overload and Maintain Ca^2+^ Homeostasis in Cells

Ca^2+^ is an endogenous messenger. Intracellular Ca^2+^ homeostasis is required to maintain and regulate cell function. Reperfusion can lead to disorders in intracellular Ca^2+^ and induce Ca^2+^ influx and disorders in the mechanisms for Ca^2+^ separation, which could induce myocardial Ca^2+^ overload. Increases in intracellular Ca^2+^ can activate endonuclease and lead to DNA breaks and apoptosis [32]. Therefore, reducing intracellular Ca^2+^ overload is crucial to protect the myocardium from MIRI.

It has been reported that TMP can significantly inhibit deactivation of K^+^-Na^+^-ATP and Ca^2+^-ATP in the membranes of myocardial cells during MIRI, which is important for the maintenance of intracellular Ca^2+^ stasis [33]. TMP can block the function of Ca^2+^ channels even more effectively than verapamil, and if it is combined with prostaglandin EI it can exert a strong synergistic protective effect in a rat model of MIRI [34–36]. TMP was found to not only block the entry of extracellular Ca^2+^ through Ca^2+^ channels but also inhibit the release of intracellular stored Ca^2+^ in vascular smooth muscle cells. TMP functioned as a true Ca^2+^ antagonist [37]. However, there are differences between* in vitro* and* in vivo* studies, and also results differ according to dose. More studies are required to obtain specificity of action [38].

Fas protein could increase intracellular Ca^2+^ overload. Li et al. reported that TMP could reduce Fas protein levels in the ischemic myocardium and moderate submicrostructure changes in myocardial cells [39]. They hypothesized that TMP has Ca^2+^ channel antagonist effects that, to some extent, protect the myocardium by reducing Ca^2+^ overload. However, illustration of the detailed mechanism requires further study.

Xia and Wu reported that TMP could potently protect Ca^2+^ transportation in isoproterenol-related myocardial ischemic injury [40]. Zhang et al. reported that low doses of TMP can reduce myocardial injury, increase the Ca^2+^-ATPase activity of myocardial mitochondria, improve cardiac function and intracellular Ca^2+^ concentrations in cardiocytes, and antagonize Ca^2+^ overload in rats with diastolic heart failure [41]. Zhou et al. reported that perfusion of Ca^2+^ and Ca^2+^ complexes in animal hearts could not induce similar myocardial injury to MIRI, especially early afterdepolarization (EAD) [42]. TMP could potently inhibit EAD, and this effect could be ameliorated by liquids containing high levels of Ca^2+^. It was considered that the effect of TMP in the treatment and prevention of MIRI was related to Ca^2+^ antagonism. The effect of TMP on attenuate calcium overload and maintain cellular calcium homeostasis was shown in Table 3.

### 2.4. Inhibit Apoptosis and Protect Myocardial Cells

Apoptosis is programmed cell death. It may occur in certain pathophysiological conditions. It is a key step of myocardial cell death in acute myocardial ischemia-reperfusion [43], the population of which decides the severity of MIRI [44].

Duan and colleagues observed that apoptosis can occur in a rat model of MIRI and that cell populations as well as myocardial pathological changes are aggravated with time delay [45]. However, this could be alleviated by using TMP, which suggested that TMP inhibits apoptosis during MIRI. Xie et al. reported that TMP could not prevent but could slow down myocardial apoptosis induced in pressure-overloaded Sprague-Dawley rats, which suggests that TMP may have a cardioprotective effect [46].

Lipopolysaccharide (LPS) can boost the gene expression of cycloxygenase-2 (COX-2), which in turn can produce a series of inflammatory mediators. TMP can significantly inhibit expression of COX-2 mRNA and protein but does not affect COX-2 activity, which suggests that TMP inhibits COX-2 at the gene level by blocking LPS signals and antagonizing LPS-induced apoptosis of myocardial cells in rats [47].

Gene regulation of apoptosis involves promotion and inhibition. Bcl-2 is one of the most important apoptosis inhibitors, and Bax is a promoter. The Bcl-2/Bax ratio is considered to be a key factor of cell activity. Liu and colleagues illustrated that TMP could significantly upregulate bcl-2 expression without affecting Bax expression, which suggests that TMP can reduce MIRI-induced apoptosis [48]. This finding was confirmed by another study. TMP not only suppressed downregulation of expression of Bcl-2, upregulation of expression of Bax, and release of mitochondrial cytochrome c to the cytosol, but also attenuated caspase-3 activation and eventually protected against H_2_O_2_-induced apoptosis [49]. Zhang and colleagues suggested that myocardial apoptosis and the Fas/FasL system are involved in the occurrence and development of myocardial ischemic injury in rats [50]. Zhang and colleagues stated that TMP may inhibit Fas/Fas L and caspase-3 levels in ischemic myocardial reperfusion and that caspase-3 is one of the factors of apoptosis [51]. That study also revealed that the Fas death receptor pathway participates in MIRI by which TMP inhibits apoptosis.

Activated STAT3 can be transmitted inside a cell and, if combined with specific DNA series, can upregulate Bcl-2 expression, thereby promoting the transcription of inducible NOS, COX-2, and manganese superoxide dismutase and inhibiting apoptosis of myocardial cells. Zhai and colleagues reported that the JAK2/STAT3 signaling pathway is involved in relieving MIRI in rats using TMP [52].

Zhao and colleagues observed the influence of ligustrazine ferulate (LF) postconditioning in a rat model of MIRI. They found that TMP and LF could accelerate the heart rate, increase left-ventricular pressure of the late systolic period, increase the rate (dP/dt max) of left-ventricular pressure rise in early systole, reduce the left-ventricular pressure of the late diastolic period, increase serum SOD activity, and reduce MDA content. These phenomena reduced myocardial infarct size, the apoptosis index and expression of Fas protein [53]. They believe that LF postconditioning can relieve MIRI in rats* in vivo*. The effect of TMP on inhibition of apoptosis was shown in Table 4.

### 2.5. Interfere with the Inflammatory Reaction and Mitigate Cell Injury

Recently, the relationship between the inflammatory reaction and MIRI has become well-established [54]. The influence of TMP on decreasing cerebral ischemia and reperfusion-induced effects on activation of production of inflammatory cells and proinflammatory mediators has been confirmed [55]. Inflammatory injury continues throughout MIRI and is activated in the ischemic period and obviously aggravated upon reperfusion [56]. Interference with neutrophils and proinflammatory factors could mean an improvement in MIRI.

Hu and colleagues found that the activity of SOD and GSH-Px in the TMP preconditioning group was promoted considerably, whereas that of MDA, LDH, creatinine kinase (CK), tumor necrosis factor- (TNF-) *α*, and interleukin- (IL-) 6 was decreased and myocardial infarct size reduced as the ST interval declined [57]. These protections are due to the increased activity of SOD, GSH-Px, and the inhibited inflammatory reaction. In MIRI, high levels of free radicals activates p38 mitogen activated protein kinase (p38MAPK) and nuclear factor kappa-light-chain-enhancer of activated B cells (NF-B), both of which induce the generation of TNF-*α* and IL-6 [58]. Shang et al. reported that TMP could decrease p38MAPK activity, inhibit the expression of TNF-*α* and IL-6, and thereby protect the myocardium [59].

Pan and Jin reported that TMP combined with LF may help to protect against MIRI in rats by reducing serum CK levels as well as inhibiting expression of E-selectin and P-selectin of endothelial cell adhesion molecules [60]. Yang and colleagues reported that TMP, LF, and their combination can alleviate MIRI through inhibition of expression of the endothelial cell adhesion molecule mRNA of E-selectin, P-selectin, and intercellular adhesion molecule- (ICAM-) 1, among which the combination group was the most prominent [61]. The effect of TMP on inhibition of inflammatory reaction was shown in Table 5.

### 2.6. Interfere with Cell Signaling Pathways

The reperfusion injury salvage kinase (RISK) signaling pathway is a type of signal-regulated kinase which includes phosphatidylinositol-3 kinase (PI3K)/protein kinase B (Akt), extracellular signal-regulated kinase (ERK1/2), protein kinase C (PKC), and protein kinase G (PKG). Interference of the RISK signaling pathway (to elicit relief from MIRI) has become a “hot” research target in recent years [62].

Phosphorylation of endothelial nitric oxide synthase (eNOS) is one of the downstream targets in the PI3K/Akt pathway [63]. As reported by Lv et al., TMP has antiapoptotic and cardioprotective effects against MIRI and acts through the PI3K/Akt pathway [64]. In addition, phosphorylation of eNOS with subsequent production of NO was found to be an important downstream effector contributing significantly to the cardioprotective effect of TMP.

*In vivo* and cell-level research has shown that PKC and Gi/o protein are involved in the protection of preconditioned myocardial cells. Liang et al. reported that PKC blockers and Gi/o protein deactivators could abrogate the protection of hypoxic preconditioning and TMP preconditioning, which suggested that the mechanism of TMP preconditioning protection was probably related to PKC and Gi/o protein [65]. Chen and colleagues reported that TMP could induce delayed cardioprotective effects by activation of PKC and extracellular signal-regulated protein kinase 1/2 signaling pathways as well as subsequent increased expression of HSP70 in rat neonatal cardiomyocytes [66].

Adiponectin acts on AdipoR1 to activate the AMPK channel to promote energy utilization in infarcted areas, maintain ATP levels, and inhibit apoptosis. C. Q. Li and Y. X. Li reported that the protection afforded by TMP in MIRI rats could be related to increased levels of adiponectin [67]. The effect of TMP on interference with cell signaling pathways was shown in Table 6.

### 2.7. Improve Function of Endothelial Cells and Protect Myocardial Cells

Ischemic reperfusion in MIRI leads to disorder in endothelial cells. Wang et al. reported that TMP could protect coronary endothelial cells, increase the NO level, and reduce the level of endothelial cells in humans to alleviate MIRI [68]. Liang and colleagues reported that ischemic preconditioning reduces levels of endothelial cells and TTXB2 after reperfusion, while that of 6-Keto-PGF_1*α*_ is increased [69]. This phenomenon could explain how preconditioning can stimulate endothelial cells to release more vasodilator substances during ischemic reperfusion to protect themselves. Those studies illustrated that TMP preconditioning could protect against injury to vascular endothelial cells due to ischemic reperfusion. Li et al. reported that the antiapoptotic effect of TMP and salvianolic acid B on rheologically induced injury to endothelial cells was likely to contribute to their efficacy [70]. The effect of TMP on improving function of endothelial cells was shown in Table 7.

## 3. Discussion and Prospects

In recent years, research into the treatment and prevention of MIRI with TMP has been fruitful. The common view is that TMP protects against MIRI by multiple mechanisms: scavenging OFRs, attenuating Ca^2+^ overload, protecting endothelial cells, inhibiting apoptosis, and affecting cytokine expression [71].

However, there are so many drawbacks in this research. The lack of multicenter, randomized, double-blind, long-term, and large-scale clinical trials, the differentiation of dose, evaluation standards, and the influence of complex agents affect the evaluation of TMP [72]. Current research remains at a simple, repeatable stage with very few insights into signal-related pathways, gene regulation, and receptor channels; more information is needed to elucidate the mechanisms involved [73]. As a key factor to determine the severity of reperfusion injury, the mitochondrial permeability transition pore is becoming a new important target in cardioprotection research. If TMP is a specific Ca^2+^ antagonist, then why does it exert different effects* in vivo* and* in vitro* and in different doses? Is the antagonism related to the severity of illness? These problems should be researched further [38]. K^+^ channels (especially the K_ATP_ channel) are closely related to the onset, development, and prevention of myocardial ischemia. K_ATP_ channel openers have been attracting much attention but have been mostly ignored by TMP researchers.

MIRI is a complicated pathophysiological process with multiple synergistic actions, and its mechanism of action has yet to be elucidated. Under the direction of traditional Chinese medicine and advanced scientific methodology, the mechanism by which TMP treats and prevents MIRI should be researched further.

## Figures and Tables

**Table 1:** Protection of mitochondria and improvement of energy metabolism during MIRI.

Pharmacological action	Methods	Reference
Protect mitochondria and improve energy metabolism	Reduce myocardial ATP delay ATP breakdown	Nordlie et al. 2006 [5]
	Protect Na^+^-K^+^-ATPase activity	Wang et al. 2003 [6]
	Stimulate synthesis of protein and RNA	Zhu et al. 2010 [7]
	Maintain the complete structure of biological membranes and myocardial fibers	Shi et al. 1998 [8]
	Reduce SDH and CCO deactivation	Wang et al. 1998 [9]
	Increase activity of Ca^2+^-ATPase and Ca^2+^-Mg^2+^-ATPase and regulate expression of the Bcl-2 gene	Wan et al. 2001 [10]

**Table 2:** Scavenge OFRs to inhibit lipid peroxidation.

Pharmacological action	Methods	Reference
Scavenge OFRs to inhibit lipid peroxidation	Prevent formation of peroxynitrites	Du et al. 2009 [15]
	Activate SOD and GSH-Px	Liu et al. 2005 [18]
	Induction of HO-1	Wang et al. 2008 [19]
	Protect SOD activity	Chen et al. 2006 [20]

**Table 3:** Attenuate calcium overload and maintain cellular calcium homeostasis.

Pharmacological action	Methods	Reference
Attenuate Ca^2+^ overload and maintain cellular Ca^2+^ homeostasis	Reduce deactivation of K^+^-Na^+^-ATP and Ca^2+^-ATP	Ruiz-Meana and García-Dorado 2009 [32]
	Reduce level of Fas protein	Xu 2003 [38]
	Inhibition of early-after-depolarization	Zhang et al. 2009 [41]

**Table 4:** Inhibition of apoptosis.

Pharmacological action	Methods	Reference
Inhibit apoptosis	Stop transmission of LPS signal	Xie et al. 2004 [46]
	Promote bcl-2 expression, increase the Bcl-2/Bax ratio	Wan et al. 2004 [47]
	Attenuate activation of caspase-3	Liu and Niu 2011 [48]
	Inhibit the intensity of Fas/Fas L	Zhang et al. 2009 [50]
	Activate the JAK2/STAT3 signal pathway	Zhang et al. 2007 [51]

**Table 5:** Interference with the inflammatory reaction.

Pharmacological action	Methods	Reference
Interference with the inflammatory reaction	Decrease activity of p38MAPK	Castaneda et al. 2003 [58]
	Inhibit the expression of E-selectin and P-selectin	Shang et al. 2008 [59]
	Inhibit ICAM-1 expression	Pan and Jin 2008 [60]

**Table 6:** Interference with cell signaling pathways.

Pharmacological action	Methods	Reference
Interfere cell signal pathway	Activate the PI3K/Akt-eNOS signaling pathway	Fulton et al. 1999 [63]
	Increase the activity of PKC and Gi/o	Jiang et al. 2012 [64]
	Increase expression of HSP70	Liang et al. 2001 [65]
	Increase adiponectin level	Chen et al. 2007 [66]

**Table 7:** Improve function of endothelial cells.

Pharmacological action	Methods	Reference
Improve endothelial cell function	Increase the level of NO and decrease the number of endothelial cells	C. Q. Li and Y. X. Li 2010 [67]
	Increase the level of 6-Keto-PGF_1*α*_ and decrease the level of TXB_2_	Wang et al. 2001 [68]

## Abbreviations

COX-2: Cyclooxygenase-2
eNOS: Endothelial nitric oxide synthase
ERK1/2: Extracellular signal-regulated kinases
GSH-Px: Glutathione peroxidase
HO-1: Heme oxygenase-1
HSP70: Heat shock protein 70
ICAM-1: Intercellular adhesion molecule-1
LDH: Lactate dehydrogenase
LF: Tetramethylpyrazine ferulate
LPS: Lipopolysaccharide
MIRI: Myocardial ischemia-reperfusion injury
NF-*κ*B: Nuclear factor kappa-light-chain-enhancer of activated B cells
OFR: Oxygen free radical
p38MAPK: p38 mitogen-activated protein kinase
RISK: Reperfusion injury salvage kinase
PI3K: Phosphatidylinositol-3 kinase
SDH: Succinate dehydrogenase
SOD: Superoxide dismutase
TMP: Tetramethylpyrazine.
